# Frontotemporal Pathology in Motor Neuron Disease Phenotypes: Insights From Neuroimaging

**DOI:** 10.3389/fneur.2021.723450

**Published:** 2021-08-16

**Authors:** Mary Clare McKenna, Philippe Corcia, Philippe Couratier, We Fong Siah, Pierre-Francois Pradat, Peter Bede

**Affiliations:** ^1^Computational Neuroimaging Group, Trinity College Dublin, Dublin, Ireland; ^2^Department of Neurology-Neurophysiology, CRMR ALS, Tours, France; ^3^UMR 1253 iBrain, University of Tours, Tours, France; ^4^LITORALS, Federation of ALS Centres: Tours-Limoges, Limoges, France; ^5^ALS Centre, Limoges University Hospital (CHU de Limoges), Limoges, France; ^6^Pitié-Salpêtrière University Hospital, Sorbonne University, Paris, France

**Keywords:** ALS, MND, PLS, MRI, PET, SMA, PMA, SBMA

## Abstract

Frontotemporal involvement has been extensively investigated in amyotrophic lateral sclerosis (ALS) but remains relatively poorly characterized in other motor neuron disease (MND) phenotypes such as primary lateral sclerosis (PLS), progressive muscular atrophy (PMA), spinal muscular atrophy (SMA), spinal bulbar muscular atrophy (SBMA), post poliomyelitis syndrome (PPS), and hereditary spastic paraplegia (HSP). This review focuses on insights from structural, metabolic, and functional neuroimaging studies that have advanced our understanding of extra-motor disease burden in these phenotypes. The imaging literature is limited in the majority of these conditions and frontotemporal involvement has been primarily evaluated by neuropsychology and post mortem studies. Existing imaging studies reveal that frontotemporal degeneration can be readily detected in ALS and PLS, varying degree of frontotemporal pathology may be captured in PMA, SBMA, and HSP, SMA exhibits cerebral involvement without regional predilection, and there is limited evidence for cerebral changes in PPS. Our review confirms the heterogeneity extra-motor pathology across the spectrum of MNDs and highlights the role of neuroimaging in characterizing anatomical patterns of disease burden *in vivo*. Despite the contribution of neuroimaging to MND research, sample size limitations, inclusion bias, attrition rates in longitudinal studies, and methodological constraints need to be carefully considered. Frontotemporal involvement is a quintessential clinical facet of MND which has important implications for screening practices, individualized management strategies, participation in clinical trials, caregiver burden, and resource allocation. The academic relevance of imaging frontotemporal pathology in MND spans from the identification of genetic variants, through the ascertainment of presymptomatic changes to the design of future epidemiology studies.

## Introduction

**Figure 1:**
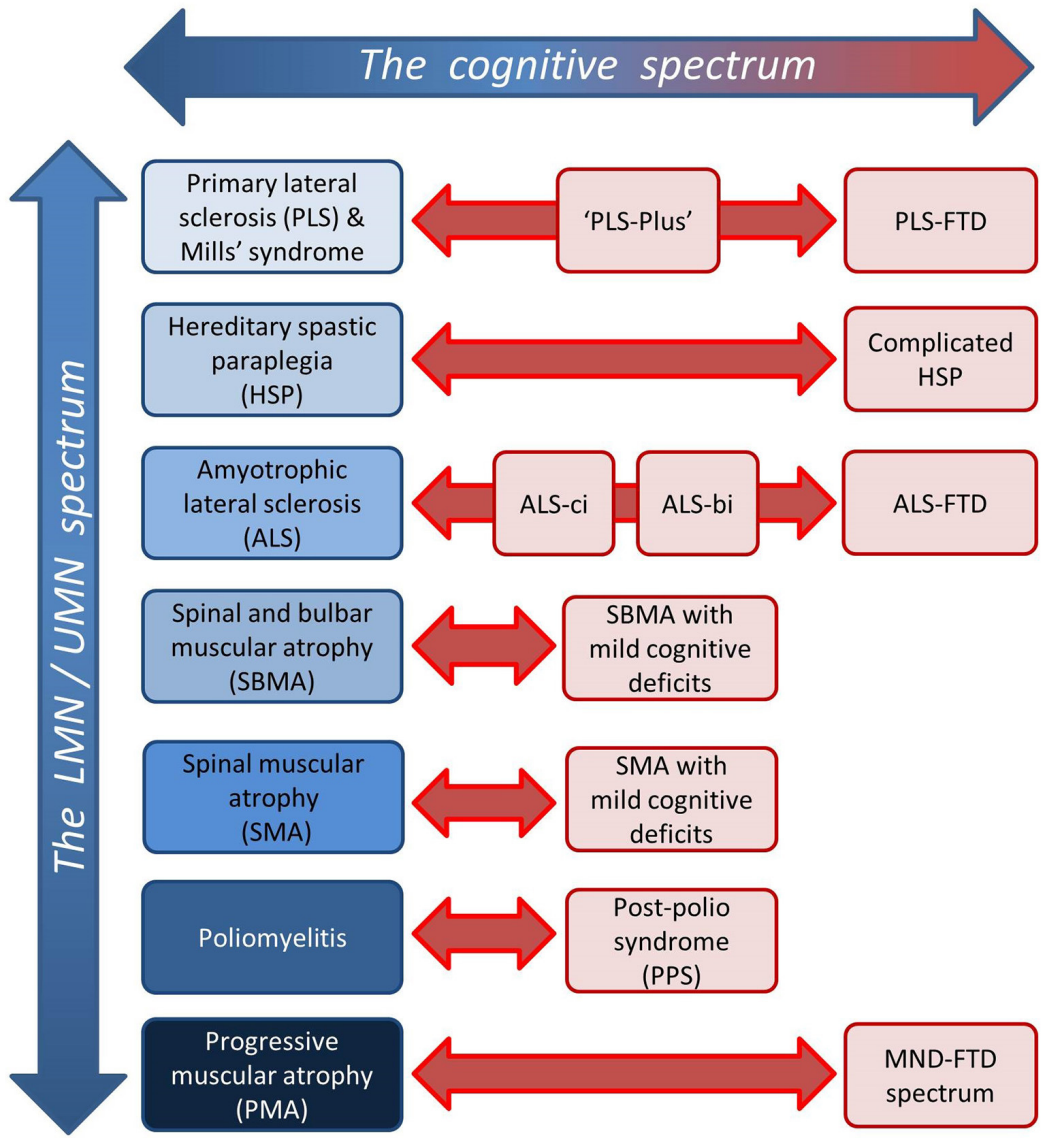
Dimensions of disease heterogeneity in MND; the spectrum of relative upper/lower motor neuron involvement and the spectrum of extra-motor manifestations.

This review explores the role of neuroimaging in characterizing frontotemporal pathology in motor neuron diseases (MNDs). While frontotemporal involvement has been extensively investigated in amyotrophic lateral sclerosis (ALS) (Table 1), it is relatively under evaluated in other MND phenotypes, such as primary lateral sclerosis (PLS) (Table 2), progressive muscular atrophy (PMA), spinal bulbar muscular atrophy (SBMA), spinal muscular atrophy (SMA), hereditary spastic paraplegia (HSP), poliomyelitis and post poliomyelitis syndrome (PPS) (Table 3). Progressive muscular atrophy, SBMA, SMA, and poliomyelitis were once regarded as pure anterior horn cell disorders, but emerging data shows that the central nervous system is more widely involved than previously thought (48, 50). Primary lateral sclerosis was traditionally considered a pure UMN condition, but extra-motor manifestations are now gradually recognized (29) (Figure 1). The ALS-FTD continuum of neurodegenerative disorders share common clinical, radiological, genetic, and pathological features (80, 81). Similar cognitive and behavioral manifestations, however, have also been described in the non-ALS MND phenotypes (82, 83). The low incidence of these slowly progressive UMN or LMN predominant disorders coupled with heterogeneous frontotemporal manifestations are all factors that may contribute to delayed or mistaken diagnoses (84–87). Caregiver burden is not only heightened by diagnostic delay, but may be exacerbated by considerable behavioral challenges (88, 89). Frontotemporal involvement may impact on entry into clinical trials and decision to participate in research studies, potentially leading to participation bias. From an academic viewpoint, there are synergistic efforts to evaluate frontotemporal disease burden using computational imaging in combination with clinical instruments. In parallel, these advances help to advance our understanding of disease pathology, propagation patterns, and the dynamics of anatomical spread. The objective of this review is to collate evidence from robust neuroimaging studies, distill emerging research trends, identify pertinent gaps in the literature, highlight clinical implications, and postulate research priorities in the evaluation of frontotemporal pathology across the spectrum of MND phenotypes.

**Table 1:** Selection of original neuroimaging research articles in ALS since 2015 with more than 30 patients.

**References**	**Study design**	**Sample size** **Study participants**	**Raw imaging data/Imaging technique**	**Assessment tools**	**Follow-up interval (months)**	**Key study findings**
Agosta et al. (1)	Cross-sectional, Prospective, Multi-center, Case-control	ALS *n* = 56 UMN phenotype *n* = 31 LMN phenotype *n* = 14	MRI Cortical thickness DTI	ALSFRS-R MMSE RCPM CET WCST WCFST RAVLT BADA HDRS FBI ALS-FTD questionnaire Phonemic and semantic fluency Digit span forwards and backwards	N/A	- Cortical thinning of the bilateral precentral gyrus, insular and cingulate cortices, and frontotemporal regions was detected in all groups. - There was involvement of the extra-motor WM tracts in the corpus callosum and frontotemporal regions. - These findings were more marked in those with cognitive or behavioral impairment.
Alshikho et al. (2)	Longitudinal, Prospective, Single-center, Case-control	ALS *n* = 53 PLS *n* = 11	MRI Cortical thickness DTI ROI analysis [^11^C]-PBR28 PET	ALSFRS-R UMNB	6-months	- In ALS, increased glia activation was detected in the precentral and paracentral gryi that correlated with areas of cortical thinning.
Alruwaili et al. (3)	Cross-sectional, Prospective, Single-center, Case-control	ALS *n* = 30	MRI VBM DTI TBSS	ALSFRS-R ACE-III FAB ALS-FTD questionnaire	N/A	- GM and WM degeneration was detected in the motor and extra motor regions in those with and without cognitive impairment. The WM alterations were more extensive in those with cognitive impairment.
Basaia et al. (4)	Cross-sectional, Prospective, Multi-center, Case-control	ALS *n* = 173 PLS *n* = 38 PMA *n* = 28	MRI DTI Resting-state fMRI Global brain network analysis Functional connectivity analysis	MMSE RAVLT Digit span forward and backward Stroop test CET WCFST WCST RCPM Phonemic and semantic fluency Italian battery for the assessment of aphasic disorders. HDRS Beck depression inventory FBI ALS-FTD Questionnaire	N/A	- In ALS, there was widespread motor and extra-motor network degeneration.
Bede et al. (5)	Cross-sectional, Prospective, Single-center, Case-control	ALS *n* = 36	MRI DTI TBSS ROI analysis	ALSFRS-R Neuropsychological assessment	N/A	- In patients with ALS without cognitive impairment, WM degeneration was detected in the cerebellum, brain stem, occipital lobes, operculum, and insula.
Bede et al. (6)	Cross-sectional, Prospective, Single-center, Case-control	ALS *n* = 70	MRI Cortical gray matter morphometry Basal ganglia morphometry DTI	ALSFRS-R		- There is GM degeneration of the anterior cingulate, orbitofrontal cortex, and mesial temporal lobes. There is also WM degeneration involving the fornix. - There is subcortical involvement of the thalamus, caudate, nucleus accumbens and hippocampus.
Bede et al. (7)	Longitudinal, Prospective, Single-center, Case-control	ALS *n* = 32	MRI VBM Cortical thickness DTI ROI analysis	ALSFRS-R	4-months 8-months	- WM degeneration was detected early with limited interval progression; GM degeneration was limited at baseline with continued progression.
Bede et al. (8)	Longitudinal, Prospective, Single-center, Case-control	ALS *n* = 100 PLS *n* = 33 FTD *n* = 30	MRI Volumetry, vertex and morphometry analyses	ALSFRS-R	4-months	- In ALS, there was progressive brainstem atrophy predominantly in medulla oblongata.
Bede et al. (9)	Longitudinal, Prospective, Single-center, Case-control	ALS *n* = 100 PLS *n* = 33 FTD *n* = 30	MRI Volumetry analyses	ALSFRS-R	4-months	- In ALS, there was progressive brainstem atrophy in the medulla oblongata and the pons.
Chipika et al. (10)	Cross-sectional, Prospective, Single-center, Case-control	ALS *n* = 100 PLS *n* = 33	MRI Volumetry analyses ROI morphometry	ALSFRS-R ECAS	N/A	- In ALS, there was involvement of the motor and sensory regions of the thalamus.
Christidi et al. (11)	Cross-sectional, Prospective, Single-center, Case-control	ALS *n* = 42	MRI DTI	ALSFRS-R RAVLT BSRT RCFT	N/A	- The involvement of WM in the frontotemporal and hippocampal regions was associated with verbal and non-verbal episodic memory test results.
Christidi et al. (12)	Cross-sectional, Prospective, Single-center, Case-control	ALS *n* = 50	MRI VBM DTI TBSS	ALSFRS-R MMSE TMT WCST RAVLT BSRT RCFT WAIS Stroop test Phonemic fluency Digit span forward and backwards	N/A	- There were motor and extra-motor GM and WM changes in non-demented cognitively-impaired ALS patients. Some of these findings were also observed in those with ALS without cognitive impairment.
Christidi et al. (13)	Cross-sectional, Prospective, Single-center, Case-control	ALS *n* = 50 AD *n* = 18	MRI Hippocampal volumetry DTI	ALSFRS-R RAVLT BSRT RCFT ALS Depression Inventory	N/A	- Patients with ALS and Alzheimer's disease have divergent hippocampal imaging signatures. - The cornu/ammonis 2/3 subfield and the hippocampus-amygdala transition area are the most affected regions in ALS.
Consonni et al. (14)	Cross-sectional, Prospective, Single-center, Case-control	ALS *n* = 48	MRI Cortical thickness	ALSFRS-R MMSE RAVLT BADA FBI Recognition memory test Neuropsychological examination of aphasia Visual object and space perception battery Digit span forward and backwards Letter fluency test Stroop test Brixton spatial anticipation test Ekman 60-faces test Story-based empathy task Dysexecutive questionnaire	N/A	- There was cortical thinning in the frontoparietal region independent of cognitive and behavioral status. - Cortical thinning involving the following regions were associated with the specific deficits: ° The Inferior frontal, temporal, cingular, and insular regions were associated with cognitive or behavioral impairments. ° The left temporal pole and insular regions were associated with language deficits.
Illán-Gala et al. (15)	Cross-sectional, Prospective, Single-center, Case-control	ALS *n* = 31 bvFTD *n* = 20	MRI Cortical thickness Cortical mean diffusivity	ALSFRS-R MMSE ECAS	N/A	- In ALS without cognitive impairment, cortical thinning was restricted to the dorsal motor cortex. In ALS with cognitive impairment, cortical thinning involved the frontoinsular and temporal regions bilaterally.
Machts et al. (16)	Cross-sectional, Prospective, Multi-center, Case-control	ALS *n* = 67	MRI Subcortical volumetry, shape, density analyses	ALSFRS-R Letter and semantic fluency test TMT Stroop test Digit span backwards FrSBe	N/A	- In ALS with or without cognitive impairment there were distinctive patterns of basal ganglia atrophy. There were structure specific correlations between imaging and neuropsychological measures.
Masuda et al. (17)	Cross-sectional, Prospective, Single-center, Case-control	ALS *n* = 51	MRI VBM DTI TBSS	MMSE FAB RCPM Alzheimer's disease assessment scale Stroop test Digit span forward and backwards Letter and semantic fluency	N/A	- In ALS with and without cognitive impairment, there were disrupted networks between the caudate and medial prefrontal or lateral orbitofrontal cortex.
Rosskopf et al. (18)	Cross-sectional, Prospective, Single-center, Case-control	ALS *n* = 140 PLS *n* = 30	MRI DTI WBSS ROI analysis	N/A	N/A	- WM degeneration involving the corticospinal tracts, corpus callosum, frontal, and brainstem regions.
Srivastava et al. (19)	Cross-sectional, Prospective, Multi-center, Case-control	ALS *n* = 65	MRS	ALSFRS-R Verbal fluency Semantic fluency Digit span forward and backwards	N/A	- There was reduced tNAA/Cr and tNAA/Cho in the prefrontal cortex. Verbal fluency, semantic fluency, and digit span forwards and backwards were associated with prefrontal tNNA/Cr.
				Digit ordering Finger and foot tapping		
Shen et al. (20)	Cross-sectional, Prospective, Multi-center, Case-control	ALS *n* = 638	MRI VBM	N/A	N/A	- There was reduction in extra-motor GM volume involving the left Rolandic operculum, inferior frontal gyrus, and superior temporal gyrus.
Westeneng et al. (21)	Longitudinal, Prospective, Single-center, Case-control	ALS *n* = 112	MRI Subcortical volumetry Subcortical shape analysis	ALSFRS-R	5.5 months	- In ALS, progressive subcortical degeneration correlated with clinical parameters.

*ACE-III, Addenbrooke's cognitive examination III; AD, Alzheimer's disease; ALS, amyotrophic lateral sclerosis; ALSFRS-R, amyotrophic lateral sclerosis functional rating scale revised; BADA, battery for the aphasic deficit analysis; bvFTD, behavioral variant frontotemporal dementia; BSRT, Babcock story recall test; CET, cognitive estimation task; DTI, diffusion tensor imaging; ECAS, Edinburgh cognitive and behavioral ALS screen; FAB, frontal assessment battery; FBI, frontal behavioral inventory; FrSBe, Frontal systems behavior scale; FTD, frontotemporal dementia; GM, gray matter; HDRS, Hamilton depression rating scale; LMN, lower motor neuron; MMSE, mini-mental state examination; MRI, magnetic resonance imaging; tNAA/Cho, total N-acetylaspartate/choline; tNAA/Cr, total N-acetylaspartate/creatine; PET, Positron emission tomography; PLS, Primary lateral sclerosis; RAVLT, Rey auditory verbal learning test; RCFT, Rey's complex figure test; ROI, region of interest; RCPM, Raven's colored progressive matrices; TBSS, tract-based spatial statistics; TMT, Trail making test; UMN, upper motor neuron; UMNB, upper motor neuron burden; VBM, voxel-based morphometry; WAIS, Wechsler adult intelligence scale; WBSS, whole brain spatial statistics; WCFST, Weigl color-form sorting test; WCST, Wisconsin card sorting test; WM, white matter*.

**Table 2:** Selection of original neuroimaging research articles in PLS.

**References**	**Study design**	**Sample size** **Study participants**	**Raw imaging data/imaging technique**	**Assessment tools**	**Follow-up interval (months)**	**Key study findings**
Agosta et al. (1)	Cross-sectional, Prospective, Multi-center, Case-control	ALS *n* = 56 UMN phenotype *n* = 31 LMN phenotype *n* = 14	MRI Cortical thickness DTI	ALSFRS-R MMSE RCPM CET WCST WCFST RAVLT BADA HDRS FBI ALS-FTD questionnaire Phonemic and semantic fluency Digit span forward and backwards	N/A	- In those with UMN phenotype, 36% had cognitive impairment and 13% had behavioral impairment - There was cortical thinning of the bilateral precentral gyrus, insular and cingulate cortices, and frontotemporal regions in all patient groups. - There was involvement of the extra-motor WM tracts of the corpus callosum and frontotemporal regions. - These findings were more marked in those with cognitive or behavioral impairment.
Alshikho et al. (2)	Longitudinal, Prospective, Single-center, Case-control	PLS *n* = 11 ALS *n* = 53	MRI Cortical thickness ROI analysis DTI [^11^C]-PBR28 PET	ALSFRS-R UMNB	6-months	- In PLS, increased glia activation was detected in the subcortical WM adjacent to the motor cortex that correlated with areas of reduced FA.
Basaia et al. (4)	Cross-sectional, Prospective, Multi-center, Case-control	ALS *n* = 173 PLS *n* = 38 PMA *n* = 28	MRI DTI Resting-state fMRI Global brain network analysis Functional connectivity analysis	MMSE RAVLT Digit span forward and backward Stroop test CET WCFST WCST RCPM Phonemic and semantic fluency Italian battery for the assessment of aphasic disorders. HDRS Beck depression inventory FBI ALS-FTD Questionnaire	N/A	- In PLS, there was widespread motor and extra-motor network degeneration.
Bede et al. (8)	Longitudinal, Prospective, Single-center, Case-control	PLS *n* = 33 ALS *n* = 100 FTD *n* = 30	MRI Volumetry, vertex and morphometry analyses	ALSFRS-R	4-months	- In PLS, there was progressive brainstem atrophy predominantly involving the medulla oblongata.
Bede et al. (9)	Longitudinal, Prospective, Single-center, Case-control	PLS *n* = 33 ALS *n* = 100 FTD *n* = 30	MRI Volumetry analysis	ALSFRS-R	4-months	- In PLS, loss of volume in medulla oblongata and pons that was more pronounced compared to ALS.
Canu et al. (22)	Cross-sectional, Prospective, Single-center, Case-control	PLS *n* = 21	MRI DTI TBSS	ALSFRS-R MMSE WCFST WCST CET RCPM RAVLT BADA HDRS FBI Phonemic and semantic fluency Digit span forward and backwards	N/A	- In those with PLS and cognitive impairment, there was WM degeneration involving the corticospinal tract, corpus callosum, cerebellum, brainstem, fornix, thalamus, and parietal lobes. These DTI metrics correlated with cognitive tests.
Chan et al. (23)	Cross-sectional, Prospective, Single-center, Case-control	PLS *n* = 18 ALS *n* = 15	MRS	N/A	N/A	- In PLS, NAA/Cr ratio was reduced in motor cortex.
Charil et al. (24)	Cross-sectional, Prospective, Single-center, Case-control	PLS *n* = 9 ALS *n* = 38	MRI MTI DWI MRS	ALSFRS-R Ashworth spasticity scale	N/A	- In PLS, NAA/Cr ratio was reduced in motor cortex and the apparent diffusion coefficient of the corticospinal tract was increased.
Chipika et al. (10)	Cross-sectional, Prospective, Single-center, Case-control	PLS *n* = 33 ALS *n* = 100	MRI Volumetry analysis ROI morphometry	N/A	N/A	- In PLS, there was a specific pattern of thalamic involvement involving motor and sensory regions and distinctive pulvinar and lateral geniculate atrophy.
Clark et al. (25)	Longitudinal, Prospective, Single-center, Case-control	PLS *n* = 18 Pre-PLS *n* = 13	MRI Volume analysis Cortical thickness DTI Resting state fMRI Task-based fMRI Finger tapping	MMSE	1–2 years	- In PLS, there was cortical thinning of the precentral gyrus. - In pre-PLS, there was progressive thinning of precentral gyrus and initial reduced motor network connectivity with some interval recovery. - The reduced FA in corpus callosum and corticospinal tracts were similar in both PLS and pre-PLS.
Fabes et al. (26)	Longitudinal, Prospective, Single-center, Case-control	PLS *n* = 6 ALS *n* = 43	MRI Quantitative FLAIR signal intensity ROI analysis	ALSFRS-R TMT	Not specified	- In PLS, highest FLAIR intensity difference in both ROIs—corticospinal tracts and corpus callosum—but there was no significant correlation with UMN signs.
Finegan et al. (27)	Cross-sectional, Prospective, Single-center, Case-control	PLS *n* = 49 ALS *n* = 100	MRI VBM DTI ROI analysis	ALSFRS-R ECAS Penn UMN score Modified ashworth spasticity scale Tapping rate	N/A	- The extra-motor profile included insular, inferior frontal and left pars opercularis pathology.
Finegan et al. (27)	Cross-sectional, Prospective, Single-center, Case-control	PLS *n* = 33 ALS *n* = 100	MRI Volumetric, morphometric, segmentation and vertex-wise analyses	ALSFRS-R Penn UMN score	N/A	- PLS was associated with considerable subcortical gray matter degeneration involving the thalamus, caudate, and hippocampus in a pattern distinct from ALS.
Finegan et al. (28)	Cross-sectional, Prospective, Single-center, Case-control	PLS *n* = 33 ALS *n* = 100	MRI Subcortical volume analysis	ALSFRS-R	N/A	- In PLS, there were specific patterns of subcortical degeneration involving the thalamus, hippocampus, and accumbens nucleus.
Finegan et al. (29)	Cross-sectional, Prospective, Single-center, Case-control	PLS *n* = 40	MRI VBM DTI ROI analysis Subcortical volume analysis	ALSFRS-R Penn UMN score Modified ashworth spasticity scale ECAS HADS FrSBe Emotional lability questionnaire	N/A	- There were GM changes involving anterior cingulate, dorsolateral prefrontal cortex, insular, opercular, orbitofrontal and bilateral mesial temporal regions. - There were WM alterations involving fornix, brainstem, temporal lobes, and cerebellum. - The volume of the thalamus, caudate, hippocampus, putamen, and accumbens nucleus were reduced. - Extra-motor clinical deficits included verbal fluency, language, apathy, and pseudobulbar affect.
Kolind et al. (30)	Longitudinal, Prospective, Single-center, Case-control	PLS *n* = 7 ALS *n* = 23	MRI mcDESPOT	ALSFRS-R ACE-III Verbal fluency TMT	7 ± 1 month	- In PLS, there were longitudinal changes in widespread cerebral myelin water fraction reductions independent of disease duration and UMN burden.
Kwan et al. (31)	Longitudinal, Prospective, Single-center, Case-control	PLS *n* = 22 ALS *n* = 21	MRI Cortical thickness Regional brain volume DTI	ALSFRS-R	2.08 ±0.3 years	- In PLS, there was reduced FA of corticospinal tracts and progressive cortical thinning that correlated with clinical progression.
Menke et al. (32)	Longitudinal, Prospective, Single-center	PLS *n* = 3 ALS *n* = 21	MRI DTI TBSS	ALSFRS-R	6-months	- In ALS and PLS, there was reduced FA in the internal capsule that correlated with rate of progression.
Meoded et al. (33)	Cross-sectional, Prospective, Single-center, Case-control	PLS *n* = 17 ALS *n* = 13	MRI VBM DTI	ALSFRS-R Delis-kaplan executive function system Mattis dementia rating scale Beck depression inventory FrSBe UCLA neuropsychiatric index	N/A	- There was WM degeneration of corpus callosum and long association tracts connecting frontotemporal areas to occipital and parietal areas that was associated with cognitive test results.
Meoded et al. (34)	Cross-sectional, Prospective, Single-center, Case-control	PLS *n* = 16	Resting state fMRI	ALSFRS-R MMSE Finger and foot tapping speed	N/A	- There was increased functional connectivity involving the cerebro-cerebellar connections to the frontotemporal and motor regions.
Mitsumoto et al. (35)	Longitudinal, Prospective, Single-center, Case-control	PLS *n* = 6 ALS *n* = 49 PMA *n* = 9	MRI DTI MRS	ALSFRS-R Finger and foot tapping Motor unit number estimation Transcranial magnetic stimulation	Every 3-months for 15-months	- In PLS, there was reduced NAA/tCr ratio in the primary motor cortex.
Paganoni et al. (36)	Cross-sectional, Prospective, Single-center, Case-control	PLS *n* = 10	MRI Cortical thickness DTI TBSS ROI analysis [^11^C]-PBR28 PET	ALSFRS-R UMNB Vital Capacity	N/A	- There were increased glia activation in the areas of cortical atrophy (pre-central gyri) and decreased FA (corpus callosum, subcortical WM adjacent to motor cortex, corticospinal tracts, superior longitudinal fasciculus).
Tartaglia et al. (37)	Cross-sectional, Prospective, Single-center, Case-control	PLS *n* = 11	MRI Volumetry analysis	ALSFRS-R	N/A	- There was reduced volume of whole brain, frontal lobe, precentral cortex, and corpus callosum.
Tu et al. (38)	Cross-sectional, Prospective, Single-center, Case-control	PLS *n* = 10 ALS *n* = 9	MRI DTI	N/A	N/A	- In PLS, there were significant alterations proximal to motor cortex in the dentato-rubro-thalamo-cortical tracts and in the cerebellar region in the dentato-rubro-thalamo-cortical and spinocerebellar tracts.
Turner et al. (39)	Cross-sectional, Prospective, Single-center, Case-control	PLS *n* = 4 ALS *n* = 34	[^11^C]-flumazenil PET	ALSFRS-R	N/A	- In PLS, the pattern of decreased binding of [^11^C]-flumazenil was similar to sporadic ALS with the exception of the relative decrease in the anterior and orbito-frontal region in sporadic ALS.
Unrath et al. (40)	Cross-sectional, Prospective, Single-center, Case-control	SBMA *n* = 20 HSP *n* = 24 PLS *n* = 25	MRI DTI	N/A	N/A	- In PLS, widespread WM degeneration involving the corticospinal tracts, corpus callosum, brainstem, and subcortical WM adjacent to motor cortices.
Van der Graaff et al. (41)	Longitudinal, Prospective, Multi-center, Case-control	PLS *n* = 12 ALS *n* = 24 PMA *n* = 12	MRS	ALSFRS-R Finger and foot tapping speed	6-months	- In PLS, decreased N-acetylaspartate, N-acetyl aspartylglutamate, and myo-inositol levels detected in primary motor cortex.
Van der Graff et al. (42)	Longitudinal Prospective, Multi-center, Case-control	ALS *n* = 12 PLS *n* = 12 PMA n-12	MRI Whole brain voxel-based analysis DTI	ALSFRS-R Finger-tapping speed Vital capacity	6-months	- In PLS, reduced FA in the subcortical WM adjacent to the primary sensory cortex, thalamus, fornix, internal capsule, and corpus callosum.
Van Weehaeghe et al. (43)	Cross-sectional, Prospective, Single-center, Case-control	PLS *n* = 10 ALS *n* = 105	[^18^]F-FDG PET	ALSFRS-R	N/A	- The pattern of regional hypometabolism was indistinguishable between ALS and PLS.
Zhai et al. (44)	Cross-sectional, Prospective, Single-center	PLS *n* = 10	MRS	Finger tapping speed Transcranial magnetic stimulation Startle testing	N/A	- NAA/Cr ratio was reduced in the motor cortex.

*ACE-III, Addenbrooke's cognitive examination III; ALS, amyotrophic lateral sclerosis; ALSFRS-R, amyotrophic lateral sclerosis functional rating scale revised; BADA, battery for the aphasic deficit analysis; CET, cognitive estimation task; DTI, diffusion tensor imaging; DWI, diffusion weighted imaging; ECAS, Edinburgh cognitive and behavioral ALS screen; FA, fractional anisotropy; FBI, frontal behavioral inventory; [^18^]F-FDG PET, fluorodeoxyglucose positron emission tomography; FLAIR, fluid-attenuated inversion recovery; fMRI, functional magnetic resonance imaging; FrSBe, frontal systems behavior scale; FTD, frontotemporal dementia; GM, gray matter; HADS, hospital anxiety and depression scale; HDRS, Hamilton depression rating scale; LMN, lower motor neuron; mcDESPOT, multi-component driven equilibrium single pulse observation; MMSE, mini-mental state examination; MRI, magnetic resonance imaging; MRS, magnetic resonance spectroscopy; MTI, magnetization transfer imaging; NAA/Cr, N-acetylaspartate/creatine; NAA/tCr, N-acetylaspartate/total creatine; PET, positron emission tomography; PLS, primary lateral sclerosis; RAVLT, Rey auditory verbal learning test; ROI, region of interest; RCPM, Raven's colored progressive matrices; TBSS, tract-based spatial statistics; TMT, trail making test; UMN, upper motor neuron; UMNB, upper motor neuron burden; VBM, voxel-based morphometry; WCFST, Weigl color-form sorting test; WCST, Wisconsin card sorting test; WM, white matter*.

**Table 3:** Selection of original neuroimaging research articles in PMA, SMA, SBMA, PPS, and HSP.

**References**	**Study design**	**Sample size** **Study participants**	**Raw imaging data/imaging technique**	**Assessment tools**	**Follow-up (months)**	**Key study findings**
**Progressive muscular atrophy**						
Agosta et al. (1)	Cross-sectional, Prospective, Multi-center, Case-control	ALS *n* = 56 UMN phenotype *n* = 31 LMN phenotype *n* = 14	MRI Cortical thickness DTI	ALSFRS-R MMSE RCPM CET WCST WCFST RAVLT BADA HDRS FBI ALS-FTD questionnaire Phonemic and semantic fluency test Digit span forwards and backwards	N/A	- 36% LMN phenotype had cognitive impairment - Cortical thinning of bilateral precentral gyrus, insular and cingulate cortices, and frontotemporal regions. - WM degeneration detected in the corpus callosum and frontotemporal tracts, including the uncinate, cingulum, and superior longitudinal fasciculi. - These findings were associated with cognitive or behavioral symptoms, especially WM changes.
Basaia et al. (4)	Cross-sectional, Prospective, Multi-center, Case-control	ALS *n* = 173 PLS *n* = 38 PMA *n* = 28	MRI DTI Resting-state fMRI Global brain network analysis Functional connectivity analysis	MMSE RAVLT Digit span forward and backward Stroop test CET WCFST WCST RCPM Phonemic and semantic fluency Italian battery for the assessment of aphasic disorders. HDRS Beck depression inventory FBI ALS-FTD Questionnaire	N/A	- In PMA, structural and functional connectomes were preserved.
Kew et al. (45)	Cross-sectional, Prospective, Single-center, Case-control	ALS *n* = 6 LMN phenotype *n* = 5	Resting-state PET Task-based PET - Motor task: moving joystick with right hand	N/A	N/A	- There was no difference in regional cerebral blood flow between LMN phenotype and controls at rest. - During motor task, there was increased activation of perisylvian areas in both ALS and LMN.
Mitsumoto et al. (35)	Longitudinal, Prospective, Single-center, Case-control	PMA *n* = 9 PLS *n* = 6 ALS *n* = 49	MRI DTI MRS	ALSFRS-R Finger and foot tapping Motor unit number estimation Transcranial magnetic stimulation	Every 3-months for 15-months	- In PMA, there was modest reduction in NAA/tCr ratio in the primary motor cortex.
Quinn et al. (46)	Cross-sectional, Prospective, Single-center	ALS *n* = 20 PMA *n* = 5	MRS	ALSFRS MMSE Letter-fluency index	N/A	- In PMA, there was greater NAA/Cr in the dorsolateral prefrontal cortex compared to ALS.
Raaphorst et al. (47)	Cross-sectional, Prospective, Multi-center, Case-control	ALS *n* = 21 PMA *n* = 18	Task-based fMRI - Letter fluency - Category fluency	ALSFRS-R UMN score HADS Dutch national adult reading test MMSE Digit span forwards and backwards WAIS-R letter-number sequencing Modified WCST Category and letter fluency Stroop test RAVLT Rivermead behavioral memory test Doors test Boston naming test Judgment of line orientation test Differential aptitude test Mental rotation task	N/A	- There was impaired letter fluency in PMA and ALS. - The letter fluency task showed lower activation in the inferior frontal gyrus in PMA and ALS. - No differences detected during category fluency.
Van der Graaff et al. (41)	Longitudinal, Prospective, Multi-center, Case-control	PMA *n* = 12 PLS *n* = 12 ALS *n* = 24	MRS	ALSFRS-R Finger and foot tapping speed	6-months	- In PMA, there was no difference in N-acetylaspartate, N-acetyl aspartylglutamate levels in the primary motor cortex at baseline. There was a significant decrease in these levels at follow-up.
Van der Graff et al. (42)	Longitudinal Prospective, Multi-center, Case-control	PMA n-12 ALS *n* = 12 PLS *n* = 12	MRI Whole brain voxel-based analysis DTI	ALSFRS-R Finger-tapping speed Vital capacity	6-months	- In PMA, there was FA reduction in extra-motor WM inferior frontal gyrus, dorsolateral prefrontal cortex, corpus callosum, hippocampus, and fornix. - DTI abnormalities were modest compared to PLS.
**Spinal muscular atrophy**						
De Borba et al. (48)	Cross-sectional, Prospective, Single-center, Case-control	SMA type III (*n* = 19) SMA type IV (*n* = 6)	MRI Cortical thickness GM volumetry	MRC muscle strength evaluation SMAFRS	N/A	The volume of cerebellar lobules VIIIB, IX and X were significantly smaller in patients with SMA.
Mendonça et al. (49)	Longitudinal, Prospective, Single-center	SMA type 0 (*n* = 3)	Qualitative MRI	N/A	1-3 years	Mild progressive global brain atrophy, predominantly WM and supratentorial structures with relative preservation of the cerebellum.
Querin et al. (50)	Cross-sectional, Prospective, Single-center, Case-control	SMA type III (*n* = 19) SMA type IV (*n* = 6)	MRI Cortical thickness DTI ROI analysis	MRC muscle strength evaluation	N/A	There was increased GM density in primary motor cortex. No white matter pathology was identified.
**Spinal and bulbar muscular atrophy (Kennedy's disease)**						
Garaci et al. (51)	Cross-sectional, Prospective, Single-center	SBMA *n* = 8	MRI DTI TBSS	ALSFRS	N/A	- Loss of WM integrity, including frontal region, that correlated with disability and disease duration.
Karitzky et al. (52)	Cross-sectional, Prospective, Single-center, Case-control	SBMA *n* = 9	MRS	N/A	N/A	- Reduced NA/Cho ratio in the brainstem and reduced NA/Cho and NAA/Cr ration in the motor regions. This did not correlate with number of CAG repeats.
Kassubek et al. (53)	Cross-sectional, Prospective, Single-center, Case-control	SBMA *n* = 18	MRI VBM	MRC muscle strength evaluation	N/A	- Subtle decreases in GM volume and extensive WM atrophy, most pronounced in the frontal areas.
Lai et al. (54)	Cross-sectional, Prospective, Single-center, Case-control	SBMA *n* = 10	[^18^]F-FDG PET	N/A	N/A	- Frontal glucose hypometabolism was detected.
Mader et al. (55)	Cross-sectional, Prospective, Single-center, Case-control	SBMA *n* = 10	MRS	N/A	N/A	- There was increased myo-inositol and macromolecular detected in the motor area. - The NAA/Cr ratio correlated with number of CAG repeats.
Pieper et al. (56)	Cross-sectional, Prospective, Single-center, Case-control	SBMA *n* = 8	MRI VBM DTI	N/A	N/A	- Subtle changes in central WM tract integrity. - The GM and WM volume was unaffected.
Unrath et al. (40)	Cross-sectional, Prospective, Single-center, Case-control	SBMA *n* = 20 HSP *n* = 24 PLS *n* = 25	MRI DTI	N/A	N/A	- In SBMA, extra-motor WM alterations detected within the limbic system or its network including precuneus, temporal lobe, and left inferior frontal lobe.
**Post-Polio syndrome**						
Bruno et al. (57)	Cross-sectional, Prospective, Single-center	PPS *n* = 22		Self-reported severity of daily fatigue and subjective problems with attention, cognition and memory	N/A	- Small discrete hyperintense signal in reticular formation, putamen, medial leminiscus or WM tracts identified in 55% of those with high levels of fatigue and in none of those with low levels of fatigue.
Demir et al. (58)	Cross-sectional, Retrospective, Single-center	PPS *n* = 11	Qualitative MRI	N/A	N/A	- Qualitative MRI were reported normal.
Shing et al. (59)	Cross-sectional, Prospective, Single-center, Case-control	PPS *n* = 36	MRI Cortical thickness Subcortical GM DTI ROI analysis	ECAS FrSBe Fatigue severity scale Piper fatigue scale HADS	N/A	- Limited cortical atrophy (cingulate gyrus and temporal pole), limited subcortical atrophy (left nucleus accumbens) and no WM degeneration were detected despite prevalent extra-motor symptoms.
Shing et al. (60)	Cross-sectional, Prospective, Single-center, Case-control	PPS *n* = 36 ALS *n* = 88	MRI Morphometry DTI TBSS ROI analysis.	MRC muscle strength evaluation ALSFRS-R	N/A	- No GM or WM degeneration detected. - Increased GM volume in cerebellar, brainstem and occipital lobe regions and increased WM integrity in pyramidal, mesial temporal and cerebellar tracts.
Trojan et al. (61)	Cross-sectional, Prospective, Single-center, Case-control	PPS *n* = 42 MS *n* = 49	MRI Normalized brain volume	Fatigue severity scale Centers for epidemiological studies depression scale	N/A	- In PPS, no significant whole brain atrophy detected. - No association between brain volume and fatigue.
**Hereditary spastic paraplegia**						
Aghakhanyan et al. (62)	Cross-sectional, Prospective, Single-center, Case-control	HSP *n* = 12	MRI DTI TBSS	N/A	N/A	- There was reduced FA with preferential involvement of frontal regions in an anteroposterior pattern.
Agosta et al. (63)	Cross-sectional, Prospective, Single-center, Case-control	HSP *n* = 44	MRI VBM DTI TBSS	SPRS MMSE Verbal and spatial span Memory prose RCFT TMT RCPM Paced Auditory Serial Addition Task Phonemic and semantic fluency Beck Depression Inventory	N/A	- The distribution of extra-motor WM degeneration involving cerebellar, limbic, corticocortical and interhemispheric regions was similar in pHSP and cHSP. It correlated with cognitive impairment.
Duning et al. (64)	Cross-sectional, Prospective, Single-center, Case-control	HSP *n* = 6	MRI Volumetry DTI	Digital symbol substitution WMS-R Digital span forward and backward TMT CWIT Letter fluency RCFT RALVT	N/A	- Structural MRI and brain volumetry were normal. - Loss of WM integrity in corticospinal tract and frontal regions in those with longer disease duration.
Erichsen et al. (65)	Cross-sectional, Prospective, Single-center, Case-control	HSP *n* = 8	MRS Volumetry ROI analysis	WAIS-R Digit symbol substitution Digit span forward and backwards Stroop test “2 and 7 selective attention test” Paced Auditory Serial Addition Test CVLT-T score Continuous Visual Memory Test RCFT Controlled Word Fluency Test	N/A	- There was reduced Cho/Cr ratio in motor cortex that was associated with some cognitive measures.
Faber et al. (66)	Cross-sectional, Prospective, Single-center, Case-control	HSP *n* = 25	MRI Cortical thickness Deep GM volumes DTI	ACE-III Neuropsychiatric Inventory SPRS	N/A	- The loss of WM integrity at the fornix and corpus callosum correlated with cognitive measures. - Basal ganglia atrophy and limited cortical thinning involving motor, limbic and parietal cortices.
França et al. (67)	Cross-sectional, Prospective, Single-center, Case-control	HSP *n* = 5	MRI VBM DTI TBSS	N/A	N/A	- Significant gray matter atrophy involving the thalamus and basal ganglia but not in the cerebral cortex. - Reduced FA involving subcortical white matter of the temporal and frontal lobes, cingulated gyrus, cuneus, striatum, corpus callosum, and brainstem.
Kassubek et al. (68)	Cross-sectional, Prospective, Single-center, Case-control	HSP *n* = 33	MRI Brain parenchymal fractions	Mini Mental Status Scale	N/A	- Global brain volume reduction involving GM and WM that was more pronounced in cHSP than pHSP.
Koritnik et al. (69)	Cross-sectional, Prospective, Single-center, Case-control	- HSP *n* = 12	Task-based fMRI Finger tapping	N/A	N/A	- There was altered cortical activation during the motor tasks.
Liao et al. (70)	Cross-sectional, Prospective, Single-center, Case-control	HSP *n* = 12	Resting state fMRI	SPRS	N/A	- The baseline neural activity and connectivity were altered in frontal regions including insula, pre-central, orbitofrontal, superior, and middle frontal gyrus.
Lindig et al. (71)	Cross-sectional, Prospective, Single-center, Case-control	HSP *n* = 15	MRI VBM DTI TBSS	SPRS	N/A	- Widespread GM and WM degeneration involving corpus callosum, thalamus, parieto-occipital, upper brainstem, cerebellum, and corticospinal tracts. - There was a correlation between DTI metrics and disease duration and severity.
Montanaro et al. (72)	Longitudinal, Prospective, Single-center, Case-control	HSP *n* = 31	MRI VBM DTI TBSS MRS	SPRS	28.9 ± 8.4 months	- There was widespread WM degeneration and reduced GM volume in right pre-frontal cortex and thalamus. - Follow-up imaging did not detect significant changes.
Oguz et al. (73)	Cross-sectional, Prospective, Single-center, Case-control	HSP *n* = 4	MRI DTI TBSS	N/A	N/A	- There were widespread WM alterations involving both the motor and extra-motor regions.
Pan et al. (74)	Cross-sectional, Prospective, Single-center	HSP *n* = 5	MRI DSI TBSS	MMSE WAIS-III Modified Ashworth Scale Tendon Reflex Grading Scale	N/A	- There was global loss of WM integrity, most marked in prefrontal and motor regions of corpus callosum.
Rezende et al. (75)	Cross-sectional, Prospective, Single-center, Case-control	HSP *n* = 11	MRI Volumetry DTI TBSS	SPRS	N/A	- No volumetry analyses abnormalities detected. - Reduced FA in corticospinal tracts and corpus callosum correlated with disease severity.
Scheuer et al. (76)	Cross-sectional, Prospective, Single-center, Case-control	HSP *n* = 18	MRI [^18^]F-FDG PET	Modified Disability Scale RH Basic Battery Proverb Interpretation Associate Learning and Retention of 15 Word Pairs Digit Span Forwards and Backwards Sentence Repetition Symbol Digit Modalities Test TMT Block Design Test Visual Gestalt Test Danish Adult Reading Test WAIS List Learning Test Recognition memory tests Street Completion Test WCST	N/A	- There was decreased regional cerebral blood flow in the left fronto-temporal cortex. In the more disabled patients, more extensive changes were noted. - Neuropsychological tests showed impaired recognition memory of faces. The PET imaging findings were not associated with severe cognitive impairment.
Stromillo et al. (77)	Cross-sectional, Prospective, Single-center, Case-control	HSP *n* = 10	MRI Normalized brain volumes MRS	MMSE 4-grade severity scale of patients' autonomy walking abilities	N/A	- Widespread structural and metabolic brain alterations. - Lower global brain volumes and diffusely decreased values in cortical regions compared to controls. - Reduced brain volume and altered NAA/Cr ratio in the corona radiata correlated with disability scores.
Tomberg et al. (78)	Cross-sectional, Prospective, Single-center, Case-control	HSP *n* = 9	Task-based fMRI Flexion and extension of the right-hand fingers and right ankle	Modified Ashworth Scale	N/A	- There was altered cortical sensorimotor network function that may reflect damage in the corticospinal tract or compensatory mechanisms.
Unrath et al. (40)	Cross-sectional, Prospective, Single-center, Case-control	SBMA *n* = 20 HSP *n* = 24 PLS *n* = 25	MRI DTI	N/A	N/A	- In HSP, extra-motor WM alterations were detected within the frontal regions and limbic system and its projectional fibers.
Warnecke et al. (79)	Cross-sectional, Prospective, Single-center	HSP *n* = 6	Qualitative MRI DTI	Neuropsychological testing	N/A	- Qualitative MRI showed cerebellar atrophy and mild frontal cerebral atrophy. There was loss of WM integrity in corticospinal tracts, frontal lobes, and midbrain. This was thought to contribute to the impaired attention and executive function.

*ACE-III, Addenbrooke's cognitive examination III; ALS, amyotrophic lateral sclerosis; ALSFRS-R, amyotrophic lateral sclerosis functional rating scale revised; BADA, battery for the aphasic deficit analysis; CET, cognitive estimation task; Cho/Cr, Choline/creatinine; CWIT, color word interference test; CVLT, California verbal learning test; DTI, diffusion tensor imaging; ECAS, Edinburgh cognitive and behavioral ALS screen; FA, fractional anisotropy; FBI, frontal behavioral inventory; [^18^]F-FDG PET, fluorodeoxyglucose positron emission tomography; fMRI, functional magnetic resonance imaging; FrSBe, frontal systems behavior scale; GM, gray matter; HADS, hospital anxiety and depression scale; HDRS, Hamilton depression rating scale; HSP, hereditary spastic paraparesis; pHSP, pure HSP; cHSP, complicated HSP; LMN, lower motor neuron; mI/Cr, myo-inositol/creatine; MMSE, mini-mental state examination; MRC, medical research council; MRI, magnetic resonance imaging; MRS, magnetic resonance spectroscopy; NAA/Cho, N-acetylaspartate/choline; NAA/Cr, N-acetylaspartate/creatine; NAA/tCr, N-acetylaspartate/total creatine; PET, positron emission tomography; PLS, primary lateral sclerosis; RAVLT, Rey auditory verbal learning test; RCFT, Rey's complex figure test; ROI, region of interest; RCPM, Raven's colored progressive matrices; SMAFRS, spinal muscular atrophy functional rating scale; SPRS, spastic paraplegia rating scale; TBSS, tract-based spatial statistics; TMT, trail making test; UMN, upper motor neuron; VBM, voxel-based morphometry; WAIS, Wechsler adult intelligence scale; WCFST, Weigl color-form sorting test; WCST, Wisconsin card sorting test; WM, white matter; WMS-R, Weshcsler memory scale-revised*.

## Methods

This is a focused review of original neuroimaging studies that investigated frontotemporal pathology in the following MND phenotypes; PLS, PMA, SBMA, SMA, PPS, HSP, and ALS. The search engines PubMed and Google Scholar were used to identify key papers. Individual MND phenotypes were searched paired with keywords “MRI,” “PET,” “brain imaging,” “neuroimaging,” or “frontotemporal.” Only articles in English were reviewed. Editorials, opinion pieces and review articles were not selected. Additional papers were considered based on the reference list of reviewed publications. One hundred and forty-two original research neuroimaging studies were identified. Given the paucity of prospective neuroimaging studies in non-ALS MNDs, case series, neuropsychology, and post mortem studies were also reviewed in these conditions. The selected articles were systematically evaluated for cohort numbers, study design, clinical assessment, imaging methods, and anatomical focus.

## Results

### Primary Lateral Sclerosis

Primary Lateral Sclerosis is an upper motor neuron disorder that typically presents with insidious spino-bulbar spasticity in adulthood (27, 90). It is often associated with pseudobulbar affect that may trigger self-imposed social isolation. Extra-motor manifestations are increasingly recognized in PLS (83, 91), occurring in a similar behavioral and cognitive profile to ALS (83), and rarely fulfilling the diagnostic criteria for FTD (83, 91). Such deficits include impaired social cognition, executive function, verbal fluency, language, or apathy (29, 82, 83, 91). The reported cases of frank FTD evolved several years after the insidious onset of UMN signs and were associated with progressive radiological frontotemporal atrophy (91). This is in line with the mounting body of neuroimaging evidence that supports widespread frontotemporal involvement in PLS (22, 29, 33).

The radiological profile of PLS varies from limited extra-motor involvement to widespread pathology (27). Structural and diffusion data revealed degenerative changes in the fornix, body of the corpus callosum, anterior cingulate, dorsolateral prefrontal, insular, opercular, orbitofrontal, and temporal regions (22, 27, 29, 33, 37, 42). Some studies have explored associations with underlying structural abnormalities focusing on apathy, impaired executive function, language, and verbal fluency deficits (29, 33, 92). Longitudinal studies have yielded inconsistent findings with regards to progressive pathology (25, 26, 30–32, 35). A case report described progressive cortical atrophy over an 8.5-year timeframe (93). These observations would suggest that contrary to ALS, longer follow-up intervals may be required in PLS to characterize radiological trajectories. Extra-motor findings in PLS are also supported by metabolic and functional imaging studies. PET imaging studies have detected prefrontal and premotor areas of hypometabolism in PLS that are almost indistinguishable from the patterns seen in ALS (39, 43, 94). Whilst primarily used in a research setting, novel PET radioligand binding studies have also demonstrated alterations beyond the motor system, in the bilateral anterior cingulate gyri, and in left superior temporal lobe (2, 36, 39, 90, 95). MR Spectroscopy in PLS has mostly focused on the evaluation of the motor rather than extra-motor regions (24, 35). Similar to ALS, it shows reduced N-acetyl aspartate/creatinine ratios (23, 24, 35, 41, 44) and increased myo-inositol/creatinine ratios (41) in the motor cortex suggestive of neuronal dysfunction and gliosis, respectively. Resting-state fMRI studies report increased functional connectivity in frontotemporal networks (34, 92) which has been associated with executive dysfunction (4, 92). Similar to ALS, increased functional connectivity is typically considered a “compensatory response” to structural degeneration (92).

The few post-mortem studies are concordant with the extra-motor radiological profile of PLS (96–103). Frontotemporal lobar degeneration has been detected with some cases revealing ubiquitin- and TDP-43-immunoreactive neuronal cytoplasmic inclusion bodies in frontotemporal and hippocampal areas. Post-mortem studies seldom have accompanying comprehensive clinical information, but when available, features of nfvPPA or bvFTD have been described (96, 102).

### Progressive Muscular Atrophy

Progressive Muscular Atrophy is a clinical diagnosis that is defined by a gradually progressive isolated lower motor neuron disorder, evolving over many years (104). Reports of extra-motor involvement are inconsistent which is further complicated by the debate on whether PMA is a distinct entity or embedded within the spectrum of ALS (105–108). There are undeniably shared clinical, radiological, and pathological features, albeit less severe compared to ALS. While the initial exclusive LMN clinical presentation distinguishes PMA from ALS, patients with PMA often later develop UMN signs (107). The cognitive profile is also strikingly similar to ALS, with varying levels of executive function, language, fluency, and memory affected (83, 109). In contrast, minimal behavioral impairment is observed, and very few patients with PMA fulfill the diagnostic criteria for FTD (83, 109).

Some imaging studies have identified radiological abnormalities in a distribution that may explain these cognitive deficits (1, 109). Structural analyses have reported loss of white matter integrity in inferior frontal, dorsolateral pre-frontal, and hippocampal regions (42). A task-based fMRI study utilizing a letter fluency task as a test of executive function showed impaired letter fluency and abnormal pre-frontal activation (47). As a counter-argument, a recent study in PMA reported preserved structural integrity with no functional connectivity alterations (4). Neither MRI spectroscopy nor PET imaging studies have identified radiological abnormalities in extra-motor regions (45, 46). It is noteworthy that a dedicated neuropsychological study failed to find a difference between patients with PMA compared to controls (110). Potential shortcomings of the study designs must be considered, including small numbers of patients and the lack of sensitivity of either the chosen task or the imaging modality (46).

The shared neuropathological hallmarks also lend support to the opinion that PMA is part of the ALS clinicopathological continuum (105–107). The pathological substrates of TDP-43 positive inclusions and occasional fused-in-sarcoma (FUS)-positive basophilic inclusions are observed in both conditions, but at a lesser burden and more limited distribution in PMA (108, 111). Post-mortem studies in PMA typically describe LMN degeneration, occasional pyramidal tract degeneration, and additional TDP-43 positive inclusions in the primary motor cortex and hippocampus even in the absence of UMN degeneration (108, 111). These findings raise the question, if in fact the results of PMA studies should be streamlined, interpreted, and analyzed under the umbrella of ALS.

### Spinal Muscular Atrophy

Spinal Muscular Atrophy is an autosomal recessive disorder that is caused by either homozygous deletions or loss of function mutations in the survival motor neuron 1 (*SMN1*) gene resulting in a deficiency of survival motor neuron (SMN) protein (112). It typically manifests as a proximal, predominantly symmetrical motor weakness. The phenotype is stratified in levels of decreasing severity from type 0 to type IV, depending on age of symptom onset and achievement of developmental milestones (112). There are preliminary signals of cerebral involvement in the more severe phenotypes, but it is not yet clear if there is preferential involvement of frontotemporal regions.

The only two cross-sectional quantitative multimodal MRI brain studies evaluated the same 25 treatment naïve adults with type III or type IV SMA initially focusing on the cerebrum and then the cerebellum (48, 50). No supratentorial cortical atrophy was detected (50), but focal cerebellar changes were noted. In the more severe clinical phenotypes, qualitative MRI brain scans have captured more dramatic findings (49, 113–115). In type 0 SMA, widespread supratentorial, and sometimes infratentorial, brain atrophy has been reported. A longitudinal case series of patients with type 0 SMA showed interval radiological abnormalities involving the thalamus and basal ganglia (49, 114). Similar radiological findings have been described in type I SMA (115). For the most part, neuropsychological studies demonstrate preserved cognition (116–119). This is with the caveat that these studies are mostly limited to children and omit the more severe clinical phenotypes (120). Some aspects of childhood development are even deemed superior compared to healthy controls (121–125). The only neuropsychological study of adults with type II or type III SMA described normal rather than superior cognitive abilities. This study reported a possible adaptive mechanism of an inverse correlation between executive function and physical ability, but the level of executive function did not exceed healthy controls (116). In contrast, there are indications of attention and executive function deficits in children with type I SMA (120, 126).

The post mortem examination of the brain is often confounded by coexistent anoxic changes (127, 128). The more severe clinical phenotypes display more widespread features of degeneration involving the cerebral cortex, thalamus, brainstem, and some cranial nerve nuclei that are congruent with ante mortem radiological abnormalities (49, 129). Most of these regions seem spared in the milder phenotypes (127, 128, 130). This has been interpreted as selective neuronal network degeneration occurring below a threshold of SMN protein, although the true clinical significance of this is unknown (129). Overall, the radiological characterization of the more severe clinical phenotypes has proven challenging because of the rarity of the condition, significant disability, and limited life expectancy. In the advent of gene therapy, there may be opportunities for future research in this cohort.

### Spinal and Bulbar Muscular Atrophy (Kennedy's Disease)

Spinal and Bulbar Muscular Atrophy, also known as Kennedy's disease, is an X-linked trinucleotide repeat disorder due to expansion of cytosine-adenine-guanine (CAG) repeat in the androgen receptor gene (131). It is a multisystem disorder that typically presents in men in their fourth decade of life with slowly progressive weakness, bulbar involvement, and muscle atrophy due to insidious lower motor neuron degeneration (132, 133). Relatively mild cognitive deficits have been consistently described (134–136). While it is a multi-system disorder, the involvement of the central nervous system has been relatively under-evaluated from a radiological viewpoint (131).

The few brain imaging studies indicate various degree of frontotemporal involvement (53, 54, 56). Quantitative MRI analyses demonstrate a spectrum of frontal gray and white matter abnormalities ranging from entirely unaffected to subtle gray matter atrophy and extensive white matter degeneration (53, 56). Widespread loss of white matter integrity has been reported in the brainstem, corticospinal tracts, and limbic system (40, 51, 56). A single PET imaging study showed hypometabolism in frontal areas (54). The results of conflicting MR spectroscopy studies highlight that subclinical neuronal dysfunction may not be detected by certain imaging protocols (52, 55). A long echo-time MR spectroscopy study demonstrated altered metabolite ratios in the brainstem and motor regions (52); however, a short echo-time MR spectroscopy study failed to reproduce these findings (55). The discrepancy in these results may be explained by the potential pitfall of artificial metabolite elevation because of either metabolite signal overlap or incorrect baseline determination in short echo-time MR spectroscopy (137). These radiological findings are complemented by consistent reports of neuropsychological dysfunction in this cohort albeit mostly at a subclinical level (134–136). Deficits may be so subtle that performance on standard tests of executive function can be normal (134, 135). Mild deficits in social cognition have also been recorded (135). In contrast, single cases of more severe frontal dysfunction have been repeatedly described (138, 139).

Most post-mortem studies in SBMA focus on cardinal spinal cord, peripheral nerve, and proximal muscle changes (133, 139, 140). The pathological examination of cerebral hemispheres is seldom reported. A post-mortem report of an SBMA patient with significant cognitive impairment demonstrated marked diffuse subcortical gliosis in the pre-frontal region, hippocampus, and the degeneration of fronto-bulbar fibers in the midbrain without accompanying cortical pathology (139). Immunohistochemical studies have shown that the pathogenic nuclear mutant AR protein is present in abundance in the central nervous system; supporting the rationale to systematically evaluate cerebral changes in future SBMA studies (141).

### Poliomyelitis and Post-polio Syndrome

Post-polio syndrome is characterized by progressive muscular weakness with or without pain, fatigue, and muscle atrophy in patients who have recovered from a distant polio infection (61). Patients often report diverse cognitive symptoms, mostly deficits in attention or memory; however objective evidence is strongly confounded by comorbid factors such as fatigue (57, 142–144). The reportedly high prevalence of extra-motor symptoms is contrasted by the relative lack of cerebral radiological abnormalities in post-polio syndrome (59).

A quantitative MRI study detected minimal cortical and subcortical atrophy, involving the cingulate gyrus, temporal pole, and left nucleus accumbens (59). These subtle changes were not appreciated in other studies (60, 61). Qualitative MRI studies either identified no abnormalities or discrete subcortical hyperintensities that were hypothesized to contribute to the disabling comorbid fatigue (57, 58, 145). Patients with post-polio syndrome frequently exhibit high levels of self-reported fatigue, apathy, and verbal fluency deficits. In the absence of widespread frontotemporal imaging abnormalities, these extra-motor symptoms are postulated to be multifactorial in origin with factors such as low mood, poor sleep, and polypharmacy all playing an additive role (59).

These observations are corroborated by historical pathological studies that demonstrate preferential involvement of the brainstem rather than the cerebrum (57, 146, 147). This is further complicated by reports of patients with a history of polio, who later develop sporadic ALS and demonstrate mixed neuropathological features including the hallmarks of both diseases (148, 149).

### Hereditary Spastic Paraplegia

Hereditary spastic paraplegias (HSPs) are a clinically and genetically heterogenous group of neurodegenerative disorders that present as progressive limb weakness and spasticity. They were traditionally divided into “pure” or “complicated” phenotypes based on the absence or presence of extra-motor involvement, respectively (63). In recent times, there has been a shift to stratify these cohorts in accordance with their genetic diagnoses (150). Interestingly, there are radiological indicators of frontotemporal dysfunction irrespective of the subgroup.

Brain imaging studies have shown a reduction in whole brain volume in both clinical phenotypes (68). In pure HSP the volume of gray matter volume is thought to be mostly preserved, whereas in complicated HSP the volume of cortical and deep gray matter may be reduced. The only longitudinal study detected no change in cerebral volume over a 2-year follow-up period (72). This is with the caveat that longer time intervals may be required to detect a significant change. Loss of white matter integrity has been identified in the corpus callosum, in the frontotemporal and parietal regions in both groups (40, 62–64, 72, 73). The severity of these findings correlate with the degree of cognitive impairment (63). Given the relative cortical sparing, cognitive deficits in these cohorts were postulated to be primarily subcortical in origin (64, 66, 67). This was supported by MR spectroscopy reports of abnormal metabolic ratios in the subcortical white matter (65, 72, 77, 151–155). PET imaging studies detected cortical hypometabolism, sometimes implicating the frontotemporal regions (76, 156–161). This was accompanied by clinical measures of frontal dysfunction (76, 161). Resting-state fMRI studies have shown altered functional connectivity involving the primary motor cortex, insula, and superior frontal gyrus (70). Task-based fMRI studies typically report abnormal activation patterns in sensorimotor areas whilst performing motor tasks (69, 78).

In the advent of genotyping, there has been a focused effort to define the radiological signatures of specific genotypes. Spastic paraplegia 4 (SPG 4) is the most common autosomal dominant HSP subtype that is characterized by widespread white matter degeneration with relatively preserved gray matter (64, 71, 75). Subclinical cognitive deficits have been described that later follow a more rapid trajectory of decline escalating in the eights decade of life (162–165). Spastic paraplegia 11 (SPG11) and spastic paraplegia 7 (SPG7) are rare autosomal recessive HSP subtypes that reveal white matter degeneration involving the frontotemporal regions amongst other features (67, 74, 79, 150, 152, 166–168). Varying degrees of cognitive deficits including attention, memory, and executive dysfunction have been described in these genotypes and others (66, 79, 169).

The few post-mortem studies corroborate the radiological descriptions of frontotemporal pathology. Autopsy reports of those with a clinical rather than genetic diagnosis must be interpreted with caution. In clinically defined cases, marked cerebral atrophy and severe gliosis of the cerebral white matter has been described sometimes preferentially involving prefrontal and frontal areas (170–172). In SPG11, widespread frontotemporal cortical degeneration has been described (85). Similar pathological observations have been reported in SPG4, in addition to widespread ubiquitin positivity (164).

### Amyotrophic Lateral Sclerosis

Amyotrophic lateral sclerosis is the most common form of MND that is characterized by progressive upper and lower motor neuron degeneration in the motor cortex, brainstem nuclei, and anterior horn of the spinal cord. It begins with progressive limb-onset or bulbar-onset muscle weakness that clinically manifests as cramps, fasciculations, muscle wasting, difficulty swallowing, or speaking before ultimately advancing to respiratory failure (173). Additional cognitive and/or behavioral impairment is universally recognized and a minority of patients with ALS also fulfill the diagnostic criteria for FTD (174).

Clinical observations are widely supported by extra-motor neuroimaging findings. Structural imaging consistently reveals frontotemporal gray and white matter degeneration (14, 15, 18, 175–188). Gray matter atrophy has been described in the anterior cingulate, insula, operculum, inferior frontal gyrus, superior temporal gyrus, cerebellum, parietal, and occipital cortex (1, 6, 7, 14, 15, 20, 179, 185, 187–191). White matter degeneration has been detected in the body of the corpus callosum, inferior longitudinal fasciculus, uncinate fasciculus, cerebellum, inferior frontal, middle temporal, superior temporal, orbitofrontal, occipital, and parietal regions (1, 5–7, 11, 14, 15, 18, 20, 175–191). These anatomical findings are often linked to structure-specific behavioral or cognitive deficits (1, 3, 11, 13–15, 186–188, 192–195), but similar patterns have been described in the absence of overt cognitive impairment (1, 17, 189, 191, 196). Extra-motor changes were initially considered to be more prominent in those with *C9orf72* genotype compared to those with sporadic ALS (187, 196), but widespread frontotemporal involvement is not unique to *C9orf72* (197). Subcortical gray matter involvement can also be readily detected in the hippocampus, amygdala, thalamus, caudate nucleus, putamen, nucleus accumbens, and globus pallidus (6, 10, 11, 13, 16, 21, 185, 189, 196, 198, 199). Progressive brainstem pathology has also been reported preferentially involving the pons and the medulla oblongata (8, 9). Structural and diffusion studies are complemented by robust metabolic and functional imaging studies. PET imaging studies have shown frontotemporal hypometabolism involving the dorsolateral prefrontal, orbitofrontal, anterior frontal, and anterior temporal areas (94, 200–202) and regional hypometabolism has been linked to cognitive deficits in ALS (201, 203, 204). PET imaging abnormalities may precede the detection of cortical atrophy (205). While in their infancy, novel PET radioligand studies highlight microglial activation in frontotemporal regions, suggestive of localized inflammatory processes (2, 206–210). MR spectroscopy detects extra-motor abnormalities, potentially before the emergence of clinical symptoms (211). It shows reduced N-acetylaspartate indicative of neuronal dysfunction in the mid-cingulate gyrus (212), dorsolateral (46, 213), ventrolateral (214), and mesial prefrontal cortices (211, 215). Sometimes these frontal lobe abnormalities are subtle (19) and may be associated with measures of executive dysfunction (19, 46, 213). Resting-state fMRI studies captured both increased and decreased functional connectivity within networks that mediate specific behavioral and cognitive functions (216–222). Task-based fMRI studies have linked these abnormal activation patterns with different facets of cognition, specifically executive function (223–225), social cognition (224, 226–230), memory (227, 231, 232), and language (233). Executive dysfunction is associated with increased activation of the right superior and inferior frontal areas (224), left superior and mid temporal gyrus and left anterior cingulate gyrus (223) and decreased activation in the left precentral gyrus (223), and dorsolateral pre-frontal cortex (223, 225, 233); impaired social cognition is associated with increased activation in the prefrontal cortex (224, 226, 228, 229), right supramarginal area (230), right posterior temporal sulcus, and decreased activation in the bilateral hippocampus (229); memory deficits are associated with increased activation in the hippocampus (231) and superior frontal gyrus (232), and decreased activation in the right pre-frontal cortex (232); and finally impaired language is associated with decreased activation patterns in the pre-frontal cortex, right cingulate gyrus, and left temporal lobes (233). For the most part there are increased (224, 226, 228–231) or co-existing (223, 225, 227, 229, 230, 232) activation patterns which suggests either loss of inhibitory dysfunction or partial compensation to overcome early functional impairment (234–236). Overall there does not seem to be a consistent compensatory or inhibitory effect which suggests that these patients may have been captured at different stages of disease. Functional studies have also been widely utilized to evaluate extra-pyramidal dysfunction in ALS (237, 238). Emerging functional modalities, such as magnetoencephalography or spectral EEG have also confirmed widespread extra-motor dysfunction and as these technologies develop they are likely to contribute important additional insights (239–243). The majority of imaging studies in ALS explored the underpinnings of the most commonly affected neuropsychological domains (233, 244), such as the substrate of verbal fluency deficits, executive dysfunction, and behavioral impairment, but with the recognition of the relatively high prevalence of impairments in social cognition, memory deficits, and of apathy, the focus of imaging studies is likely to gradually shift (12, 245–249). Imaging changes in ALS are typically solely interpreted based on genetic and clinical profiles, and seldom correlated with other markers such biofluid markers (250–252). The radiological patterns identified by various imaging studies are largely congruent with the distribution of pathological TDP-43 (pTDP-43) aggregates in extra-motor brain regions (253–257). Patients with ALS-FTD are thought to carry increased extra-motor pTDP-43 burden compared to patients without cognitive impairment (256). A study of patients with cognitive impairment revealed correlations between regional pTDP-43 load and executive, language and fluency deficits (253).

## Discussion

**Figure 2:**
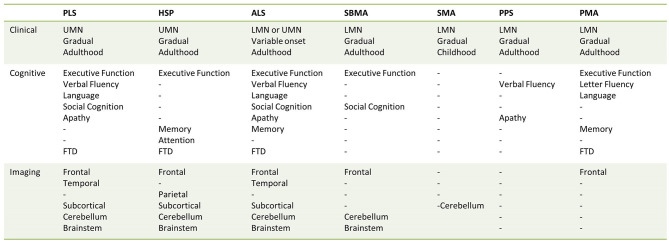
Anatomical vulnerability and clinical domains affected across the spectrum of motor neuron diseases.

This review collates evidence of radiological frontotemporal involvement in common MND phenotypes. Existing neuroimaging studies suggest that frontotemporal degeneration may be readily detected in ALS and PLS; a varying degree of frontotemporal pathology may be captured in PMA, SBMA, and HSP. Cerebral involvement without regional predilection may be exhibited in the more severe clinical phenotypes of SMA; and there is limited evidence for cerebral changes in PPS (Figure 2, Table 4). These radiological features may precede clinical symptoms, and longitudinal studies often capture gradual progression. Imaging studies in MND suffer from considerable inclusion bias because of disease-specific factors. Patients with significant apathy, motor disability, respiratory compromise, or sialorrhoea are less likely to participate or return for follow-up imaging.

**Table 4:** An overview of preferential anatomical involvement in MND phenotypes.

**PLS**	Gray matter	Primary motor cortex and precentral gyrus Prefrontal cortex and inferior frontal gyrus—insular, opercular, and orbitofrontal regions Mesial temporal lobe Anterior cingulate cortex Cerebellum
	White matter	Corticospinal tracts Corpus callosum Fornix Superior longitudinal fasciculus Brainstem—pons, medulla Cerebellum
	Subcortical	Nucleus accumbens Thalamus Hippocampus
**PMA**	Gray matter	Primary motor cortex and precentral gyrus Prefrontal cortex and inferior frontal gyrus—insular regions
	White matter	Corticospinal tracts Corpus callosum Fornix Superior longitudinal fasciculus Uncinate fasciculus
	Subcortical	Hippocampus
**SMA**	Gray matter	Global without regional predilection in severe cases Cerebellum
	White matter	–
	Subcortical	–
**SBMA**	Gray matter	Frontal lobes (subtle)
	White matter	Corticospinal tracts Inferior frontal Brainstem—midbrain Cerebellum
	Subcortical	–
**PPS**	Gray matter	Cingulate gyrus (subtle) Temporal pole (subtle)
	White matter	–
	Subcortical	Nucleus accumbens
**HSP**	Gray matter	Primary motor cortex Limbic Parietal Cerebellum
	White matter	Corticospinal tracts Corpus callosum Frontal Parietal-occipital Brainstem Cerebellum
	Subcortical	Thalamus Basal ganglia
**ALS**	Gray matter	Primary motor cortex and precentral gyrus Prefrontal cortex and inferior frontal gyrus—insular, opercular, and orbitofrontal regions Mesial temporal lobe Anterior cingulate cortex Parietal Occipital Cerebellum
	White matter	Corticospinal tracts
		Corpus callosum Arcuate fasciculus Inferior longitudinal fasciculus Uncinate fasciculus Fornix Brainstem Cerebellum
	Subcortical	Thalamus Hippocampus Amygdala Caudate nucleus Putamen Nucleus accumbens Globus pallidus

In clinical practice, the wide spectrum of frontotemporal manifestations in ALS are already incorporated in the clinical diagnostic criteria (174). It is anticipated that these features will be a fundamental part of future revisions, in conjunction with supportive neuroimaging data (258). Despite implications for survival (259), clinical staging systems of ALS have omitted to include a cognitive facet thus far (260, 261). These observations have also not yet translated into the diagnostic criteria of other MND phenotypes. While such deficits are increasingly recognized in PLS, they are deemed too infrequent to be included in the core clinical features (262). The link between FTD and other rare MND phenotypes may have important implications for everyday clinical practice, particularly given that many non-ALS MND phenotypes are associated with longer survival than ALS (263). The awareness of possible frontotemporal dysfunction may prompt the use of neuropsychological screening tests in the routine evaluation of these patients. Validated, disease-specific screening tools are preferred to generic instruments, and these are available in ALS (264, 265). Several of these are adapted to motor disability and dysarthria, and interrogate domains commonly affected in ALS. It is worth noting that patients with predominant frontotemporal cognitive deficits should be screened for incipient motor deficits (266). The early recognition of neuropsychological deficits is crucial for individualized patient care including: the appraisal of decision-making capacity, caregiver support, resource allocation, and the anticipation of management challenges (89). It may also allow clinicians to consider pharmacological and non-pharmacological interventions such as cognitive or behavioral rehabilitation. In the context of FTD, this is primarily focused on developing compensatory skills for adapting to functional impairments with the lowest level of assistance required. For example, an electronic device calendar is a daily planning tool that can be used to establish routines and set reminders to initiate activities such as taking medications (267). There is also an evolving interest in early language therapy interventions (268, 269). The education of caregivers is crucial to identify unmet needs of the patient that may trigger behavioral problems. These measures have proven to be beneficial to both the patient and their caregivers (267).

In tandem, technological innovations have enriched the supportive radiological data. High-field MRI generates better quality images and acceleration techniques enable shorter data acquisition that may be better tolerated by patients. Quantitative MRI analyses using validated computational pipelines and reliance on robust comparative, correlative, and classifier models enhance the clinical interpretation of vast imaging datasets (270). The advent of structural and functional connectivity studies have ignited interest in the concept of disease-specific selective network degeneration rather than the emphasis on focal pathology (271). These methods have proven particularly useful to differentiate clinical phenotypes and map longitudinal changes in neurodegenerative disorders (7, 271). Novel MRI pulse sequences, non-Gaussian diffusion models such as DKI or NODDI, quantitative susceptibility mapping, and multi-voxel spectroscopy are just some of the promising new tools enriching our armamentarium of imaging tools (272–274). While these methods continue to be tested in the research community they have not been implemented in routine clinical radiology protocols (275).

Frontotemporal involvement across the spectrum of MND phenotypes has important implications for clinical trials. It invites the opportunity for the development of radiological biomarkers that quantify and track frontotemporal involvement (276). Pioneering gene therapy trials have primarily focused on clinical outcome measures such as motor milestones, requiring artificial ventilation, and survival (277–279). This is also relevant because there has been interest in developing adjunctive interventions such as transcranial or neuromuscular magnetic stimulation (280, 281). These methods are not only applicable to symptomatic patients but also to those in the pre-symptomatic stages of their disease. In ALS, genotype-specific radiological alterations have been detected in pre-symptomatic carriers of pathogenic mutations decades before the onset of clinical symptoms (282–284). Awareness of associated behavioral and cognitive impairment allows for due preparation and adaption of study designs if required (285).

As we begin to incorporate these developments into clinical practice and clinical trial designs, there are pressing academic questions to be elucidated. First and foremost, it is uncertain if the motor or extra-motor changes evolve in sequence or in parallel across all phenotypes. This topic is probably best explored in ALS where there is a unique opportunity to study the pre-symptomatic phase in carriers of pathogenic *C9orf72* repeat expansions. In this cohort, radiological co-existence of motor and extra-motor involvement has been consistently described (282–286). Overall, the topography of radiological alterations is largely similar but less marked than what is described in symptomatic cases. It is unclear if the initial pattern dictates the ultimate clinical phenotype given that both FTD and ALS may have co-existent subclinical motor and extra-motor manifestations (284, 287, 288). It is also unclear whether these findings solely represent early neurodegenerative changes; some postulate that they capture a developmental abnormality (284). From a clinical perspective, early cognitive deficits have been described in pre-symptomatic carriers of *C9orf72* expansion before the phenotype is defined (282–284, 286). The notion of cognitive reserve has been increasingly evaluated in ALS which may impact on the sequence of symptom manifestation. It suggests that those with a high level of cognitive reserve, often proxied with educational attainment, require a greater degree of brain pathology to meet the threshold for clinical symptoms (289). This concept has been investigated in greater detail in FTD, but similar themes are also emerging in ALS. The level of cognitive reserve appears to predict cognitive performance and the degree of brain imaging abnormalities (290–292). These observations suggest that patient-specific factors influence the chronology of clinically evident symptoms. Some argue that the debate of whether extra-motor or motor symptoms emerge first in ALS is antiquated and that cognition and motor function are inseparably intertwined (293). It is hypothesized that the selective deficit in action words and verb processing detected in patients with ALS is in fact a cognitive manifestation of motor dysfunction (293). Although some disagree and consider it to be a feature of executive dysfunction (294). Task-based fMRI studies in healthy controls have consistently shown that reading action words activates areas along the motor strip that were responsible for conducting these movements (295). In ALS, action observation and motor imagery are routinely utilized in fMRI studies to compensate for motor disability (296).

Cognitive deficits in specific domains have been linked to the degeneration of single structures in MND (1, 14, 16, 33, 186, 188, 244). Often there is frank dissociation between cognitive and radiological findings (59, 63), but a reporting bias for confirmed associations prevents the gauging of this occurrence. Correlation analyses in ALS linked apathy to anterior cingulate and accumbens nucleus degeneration (193–195), and memory impairment to hippocampal degeneration (13). Linking cognitive deficits to single structures however may be a reductionist approach, which overlooks the role of complex cortico-subcortical networks in mediating cognitive functions (297). Accordingly, the underpinnings of neuropsychological deficits are probably best evaluated at a circuitry-integrity level instead of seeking associations with focal structures (271). Traditional structural measures are increasingly complemented by connectivity metrics to appraise the integrity of functional circuits (240, 242). The selective vulnerability of functional networks is thought to drive cardinal manifestations of neurodegenerative conditions (271). It may or may not be associated with focal atrophy of crucial nodes within these networks (298).

There are stereotyped shortcomings in the current literature that remain to be addressed. First, the low incidence of these conditions leads to small sample size despite multi-center collaborations. Second, while case-control study designs are often used to evaluate these rare disorders, this cross-sectional approach is suboptimal to characterize dynamically evolving processes. Furthermore, the indolent progression of the non-ALS MND phenotypes may require relatively long follow-up intervals to detect progressive radiological changes (72). Third, co-existing neurodegenerative disorders are potential confounders, such as behavioral variant Alzheimer's dementia. To account for this, the use of serum or cerebrospinal fluid biomarkers should be considered in future study methods to enhance diagnostic certainty. Fourth, there is a scarcity of pre-symptomatic studies and often these cohorts are not followed longitudinally until phenoconversion. Fifth, the diagnostic criteria are not well-defined in some MNDs (104). The diagnosis of “definite PLS” requires a symptom duration of at least 4 years which may further limit the number of patients available for recruitment (28). Sixth, imaging studies often concentrate on supratentorial cortical regions, overlooking the contribution of subcortical and cerebellar pathology to cognitive and behavioral manifestations. The sensitivity limitations of single imaging modalities are seldom acknowledged. Subtle abnormalities may not be detected, considerable neuronal loss may ensue before it becomes radiologically evident. Seventh, the practical implications of cognitive deficits need to be specifically investigated. The presence of cognitive impairment in ALS is considered a negative prognostic indicator that is associated with increased caregiver burden, reduced quality of life, and reduced survival; whereas the implications of cognitive impairment in other MND phenotypes is woefully under-evaluated despite their markedly longer survival (259). Finally, there is a disappointing lack of post-mortem validation of radiological findings. This is further complicated by the inherent bias of the pathological literature to favor atypical cases that are unlikely to represent the true hallmarks of these conditions.

This paper offers an overview of imaging efforts across the spectrum of MNDs to investigate frontotemporal disease expansion. It highlights the disproportionate emphasis on ALS, which offers valuable lessons to conduct similar studies in other MND phenotypes. Radiological observations highlight the rationale for routine screening for frontotemporal dysfunction to inform individualized patient care. The quality of the data may be enhanced by using multiparametric imaging protocols, longitudinal study designs, and the inclusion of pre-symptomatic cohorts where possible. The opportunity for international collaborations through carefully harmonized protocols should be explored to maximize the number of study participants in low incidence phenotypes.

## Conclusions

In contrast to ALS, the quantitative characterization of frontotemporal disease burden in non-ALS MND phenotypes remains under investigated. The nuanced evaluation of frontotemporal dysfunction across the entire spectrum of MNDs has important pragmatic implications for individualized clinical care, caregiver support, clinical trial designs, and more broadly, for our understanding of disease biology.

## Author Contributions

Manuscript drafted by MM and PB. Review for intellectual content by PCor, PCou, WS, and P-FP. All authors contributed to the article and approved the submitted version.

## Conflict of Interest

The authors declare that the research was conducted in the absence of any commercial or financial relationships that could be construed as a potential conflict of interest.

## Publisher's Note

All claims expressed in this article are solely those of the authors and do not necessarily represent those of their affiliated organizations, or those of the publisher, the editors and the reviewers. Any product that may be evaluated in this article, or claim that may be made by its manufacturer, is not guaranteed or endorsed by the publisher.
